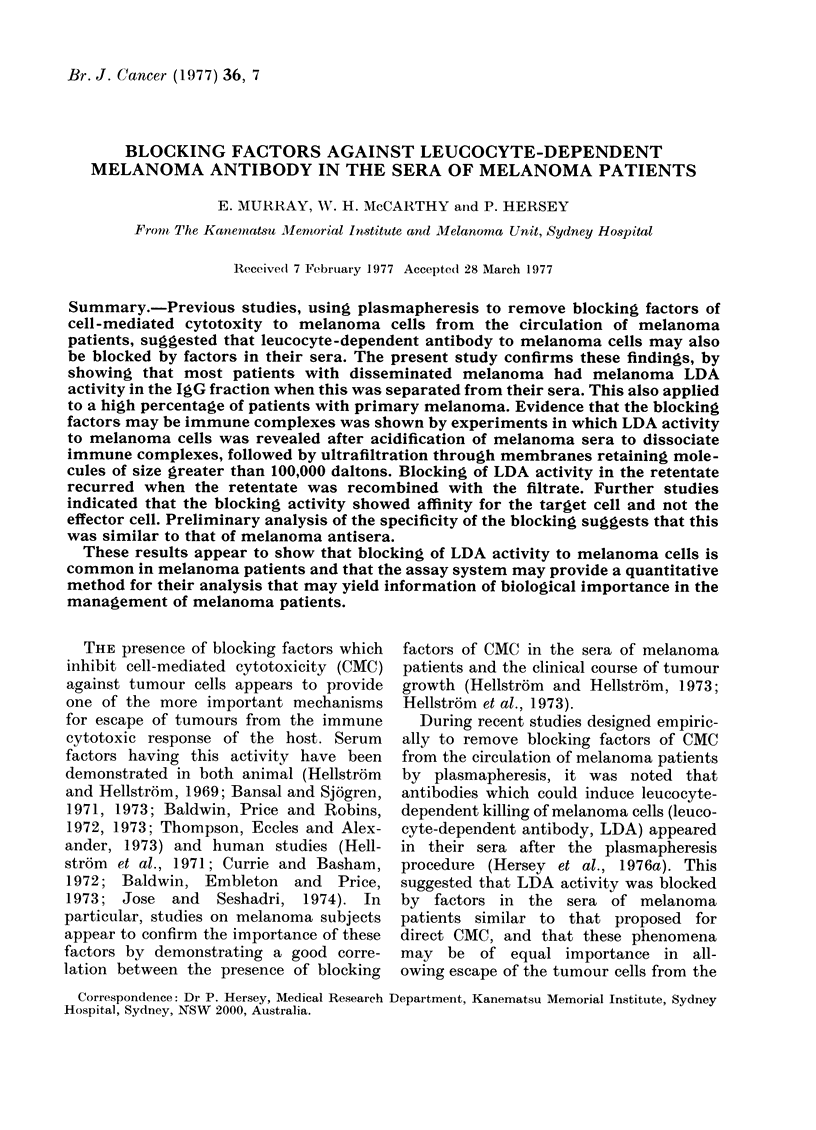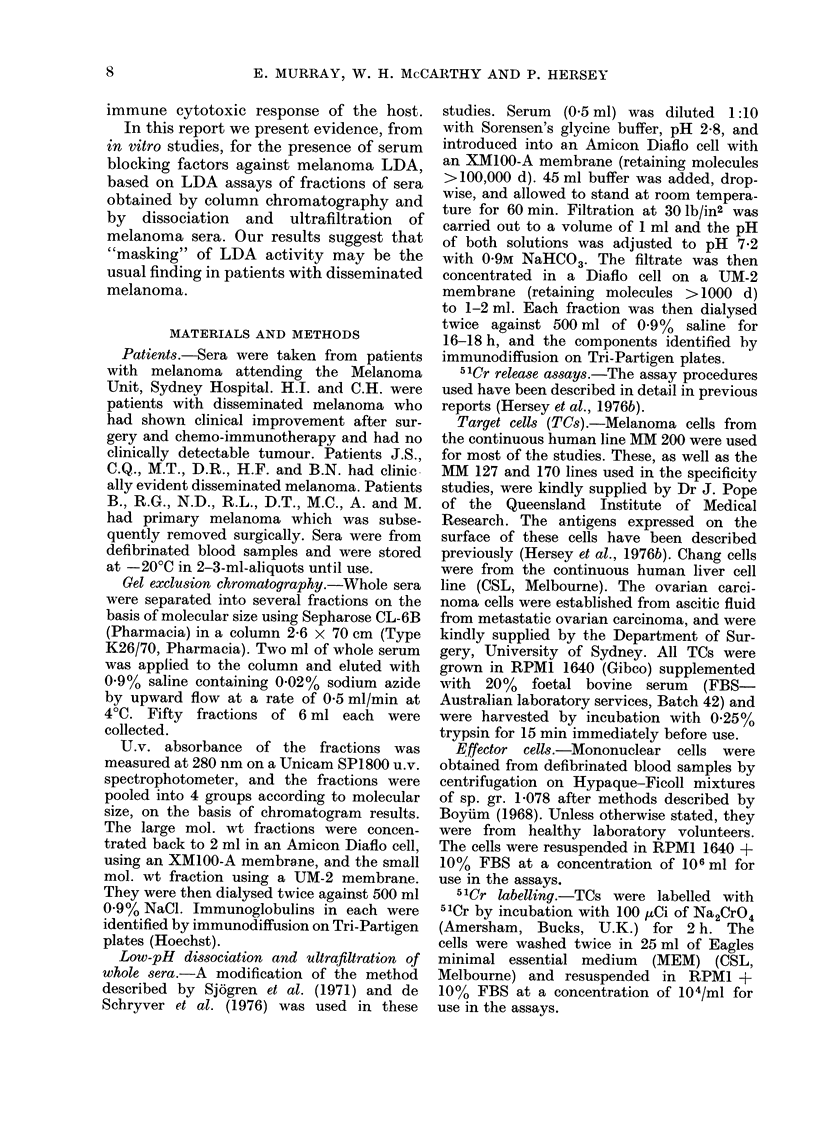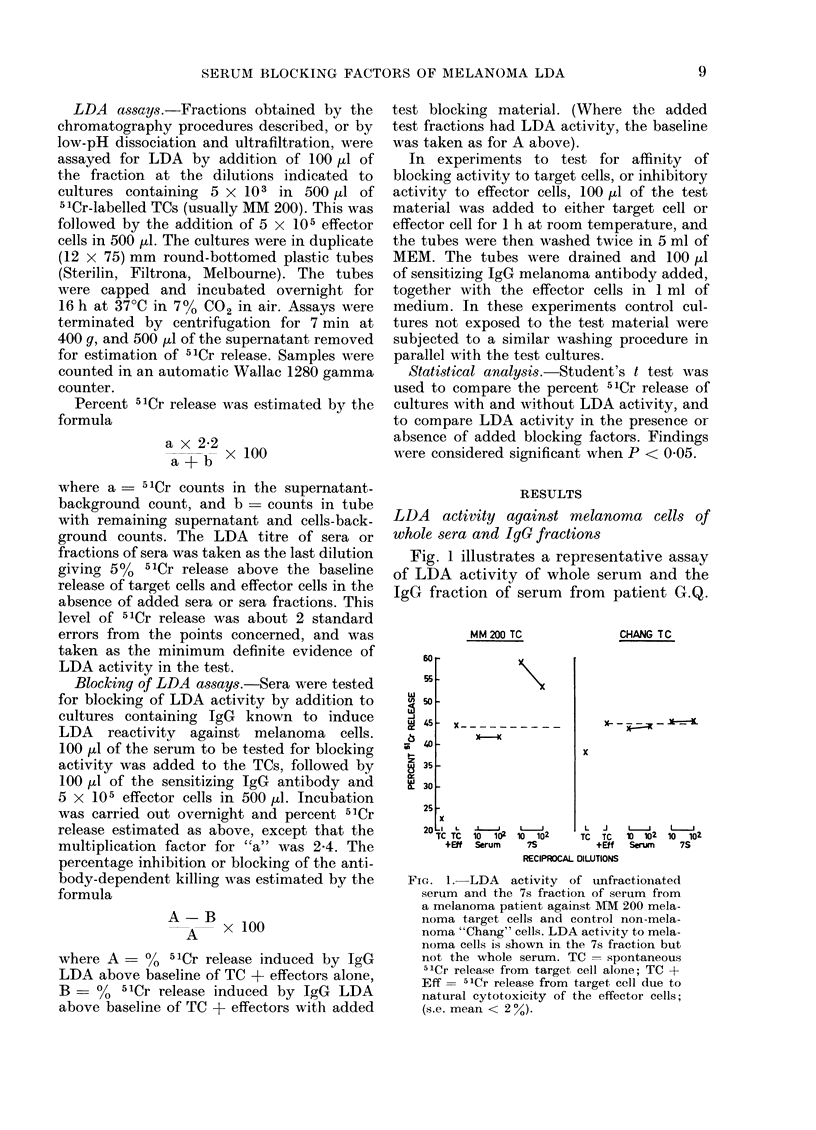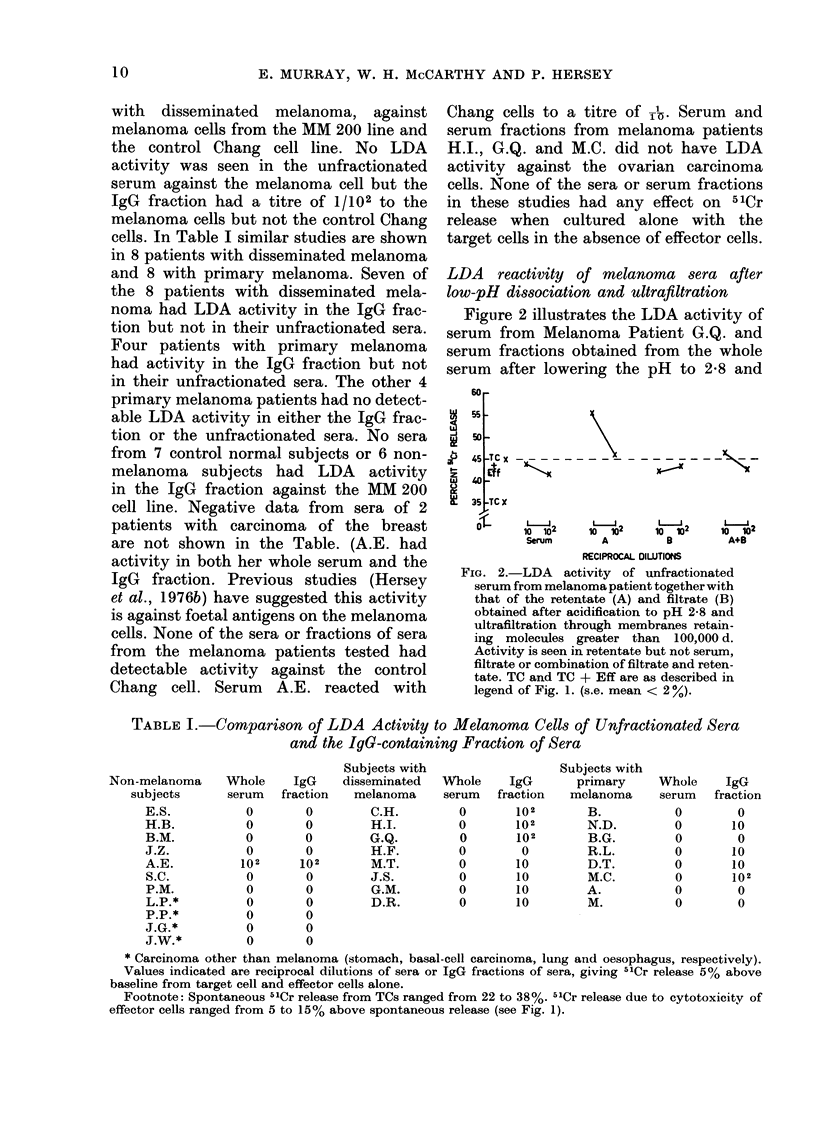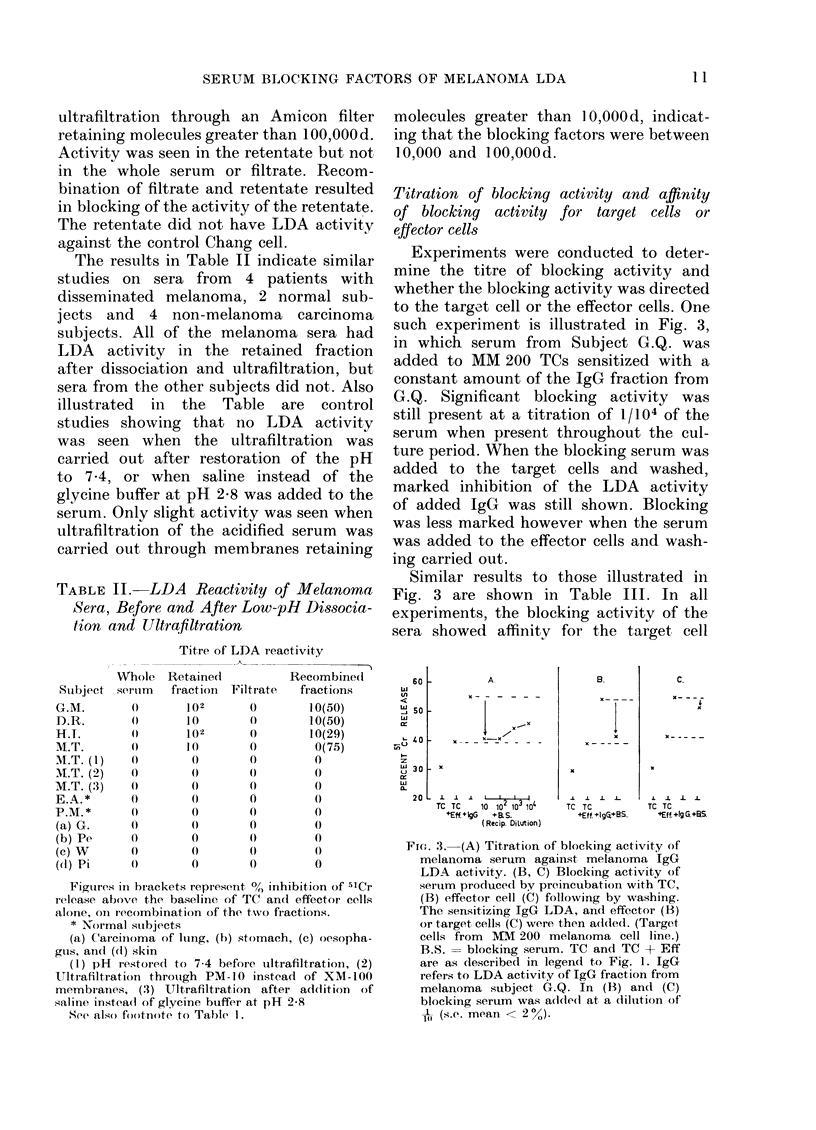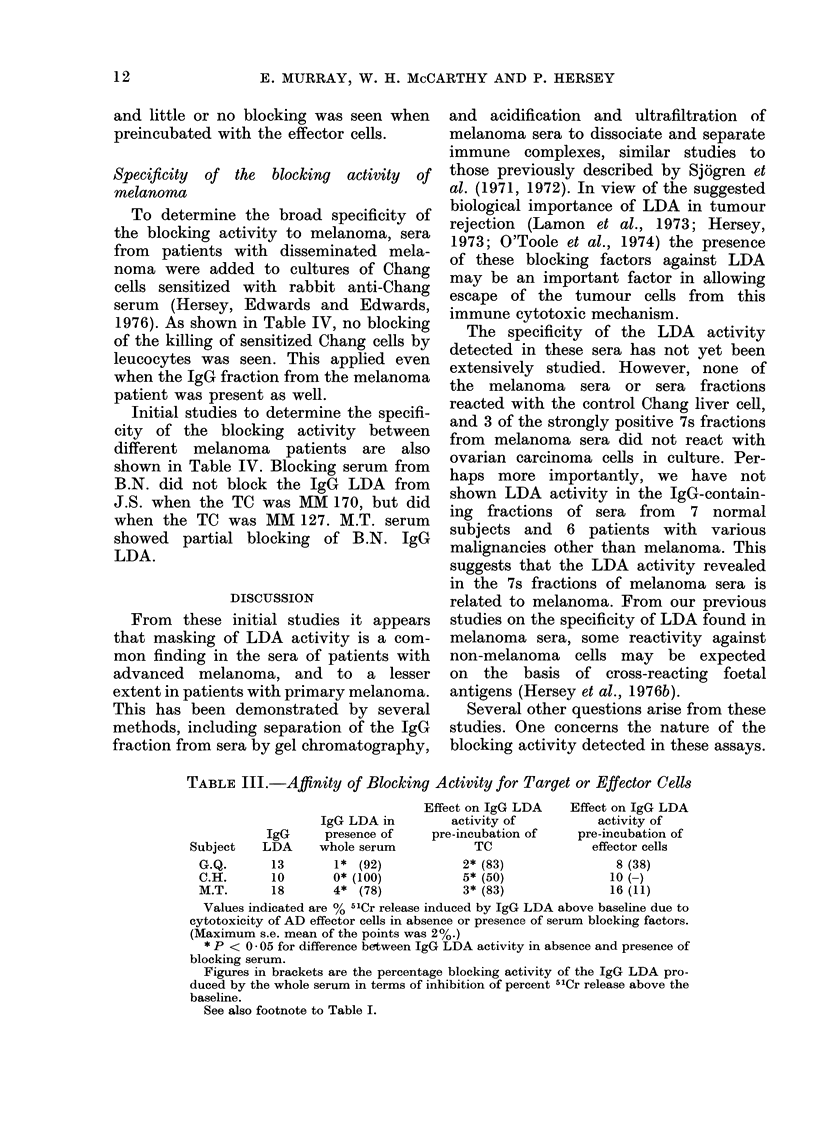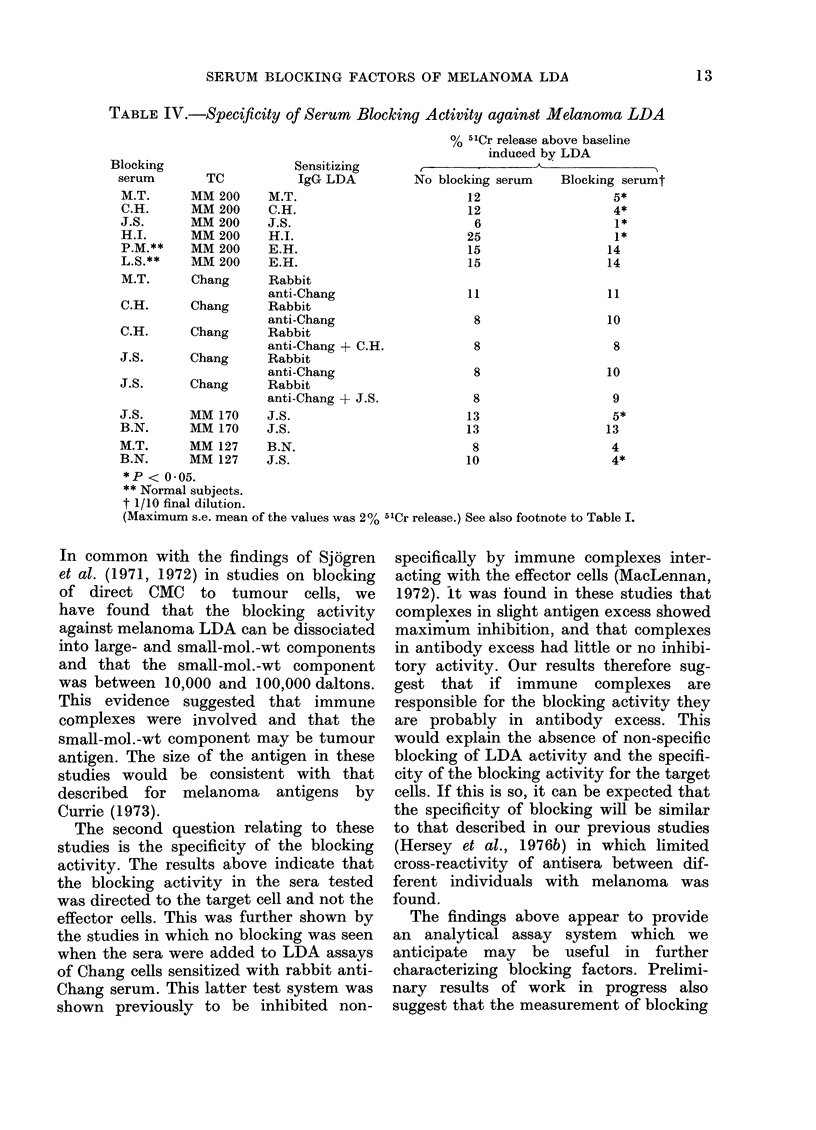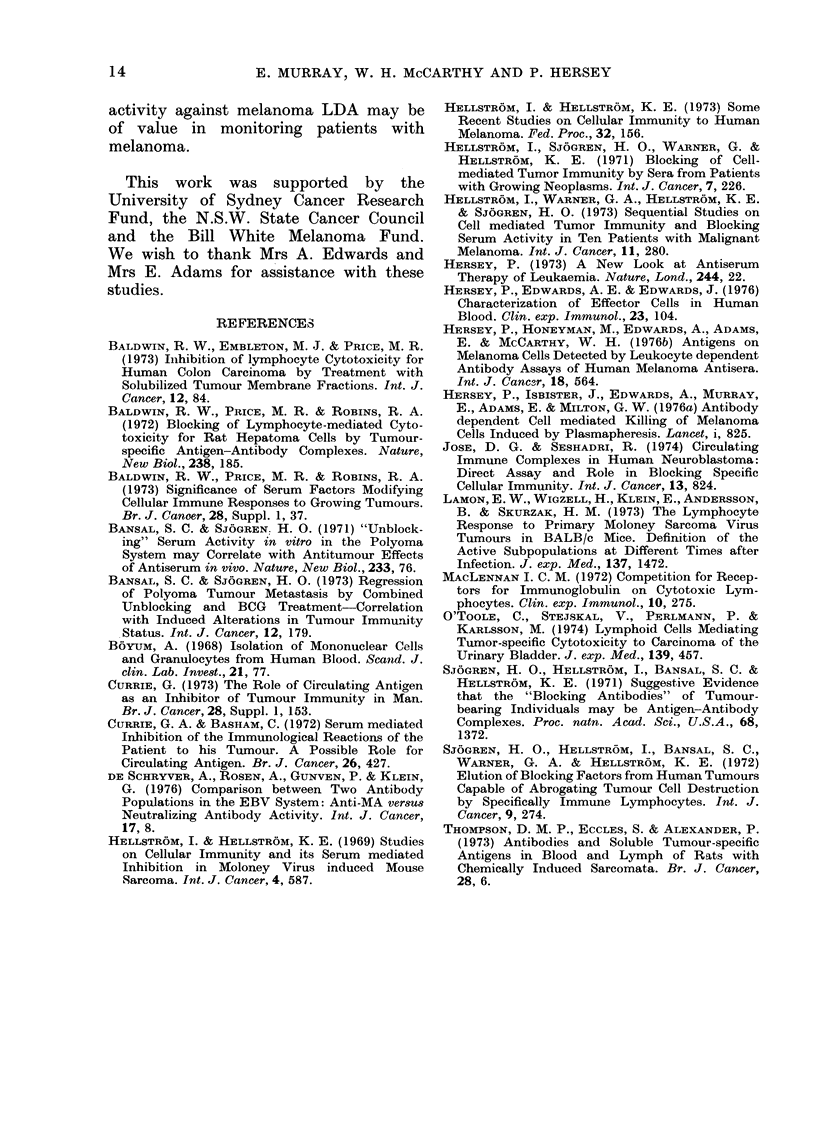# Blocking factors against leucocyte-dependent melanoma antibody in the sera of melanoma patients.

**DOI:** 10.1038/bjc.1977.148

**Published:** 1977-07

**Authors:** E. Murray, W. H. McCarthy, P. Hersey

## Abstract

Previous studies, using plasmapheresis to remove blocking factors of cell-mediated cytotoxicity to melanoma cells from the circulation of melanoma patients, suggested that leucocyte-dependent antibody to melanoma cells may also be blocked by factors in their sera. The present study confirms these findings, by showing that most patients with disseminated melanoma had melanoma LDA activity in the IgG fraction when this was separated from their sera. This also applied to a high percentage of patients with primary melanoma. Evidence that the blocking factors may be immune complexes was shown by experiments in which LDA activity to melanoma cells was revealed after acidification of melanoma sera to dissociate immune complexes, followed by ultrafiltration through membranes retaining molecules of size greater than 100,000 daltons. Blocking of LDA activity in the retentate recurred when the retentate was recombined with the filtrate. Further studies indicated that the blocking activity showed affinity for the target cell and not the effector cell. Preliminary analysis of the specificity of the blocking suggests that this was similar to that of melanoma antisera. These results appear to show that blocking of LDA activity to melanoma cells is common in melanoma patients and that the assay system may provide a quantitative method for their analysis that may yield information of biological importance in the management of melanoma patients.


Br. J. Cancer (1977) 36, 7

### BLOCKING FACTORS AGAINST LEUCOCYTE-DEPENDENT MELANOMA ANTIBODY IN THE SERA OF MELANOMA PATIENTS

E. MURRAY, W. H. McCARTHY and P. HERSEY

*From The Kanematsu Memorial Institute and Melanoma Unit, Sydney Hospital*

Received 7 February 1977 Accepted 28 March 1977

**Summary.—Previous studies, using plasmapheresis to remove blocking factors of cell-mediated cytotoxity to melanoma cells from the circulation of melanoma patients, suggested that leucocyte-dependent antibody to melanoma cells may also be blocked by factors in their sera. The present study confirms these findings, by showing that most patients with disseminated melanoma had melanoma LDA activity in the IgG fraction when this was separated from their sera. This also applied to a high percentage of patients with primary melanoma. Evidence that the blocking factors may be immune complexes was shown by experiments in which LDA activity to melanoma cells was revealed after acidification of melanoma sera to dissociate immune complexes, followed by ultrafiltration through membranes retaining molecules of size greater than 100,000 daltons. Blocking of LDA activity in the retentate recurred when the retentate was recombined with the filtrate. Further studies indicated that the blocking activity showed affinity for the target cell and not the effector cell. Preliminary analysis of the specificity of the blocking suggests that this was similar to that of melanoma antisera.**

**These results appear to show that blocking of LDA activity to melanoma cells is common in melanoma patients and that the assay system may provide a quantitative method for their analysis that may yield information of biological importance in the management of melanoma patients.**

THE presence of blocking factors which inhibit cell-mediated cytotoxicity (CMC) against tumour cells appears to provide one of the more important mechanisms for escape of tumours from the immune cytotoxic response of the host. Serum factors having this activity have been demonstrated in both animal (Hellström and Hellström, 1969; Bansal and Sjögren, 1971, 1973; Baldwin, Price and Robins, 1972, 1973; Thompson, Eccles and Alexander, 1973) and human studies (Hellström *et al.*, 1971; Currie and Basham, 1972; Baldwin, Embleton and Price, 1973; Jose and Seshadri, 1974). In particular, studies on melanoma subjects appear to confirm the importance of these factors by demonstrating a good correlation between the presence of blocking factors of CMC in the sera of melanoma patients and the clinical course of tumour growth (Hellström and Hellström, 1973; Hellström *et al.*, 1973).

During recent studies designed empirically to remove blocking factors of CMC from the circulation of melanoma patients by plasmapheresis, it was noted that antibodies which could induce leucocyte-dependent killing of melanoma cells (leucocyte-dependent antibody, LDA) appeared in their sera after the plasmapheresis procedure (Hersey *et al.*, 1976*a*). This suggested that LDA activity was blocked by factors in the sera of melanoma patients similar to that proposed for direct CMC, and that these phenomena may be of equal importance in allowing escape of the tumour cells from the

Correspondence: Dr P. Hersey, Medical Research Department, Kanematsu Memorial Institute, Sydney Hospital, Sydney, NSW 2000, Australia.

immune cytotoxic response of the host.

In this report we present evidence, from *in vitro* studies, for the presence of serum blocking factors against melanoma LDA, based on LDA assays of fractions of sera obtained by column chromatography and by dissociation and ultrafiltration of melanoma sera. Our results suggest that "masking" of LDA activity may be the usual finding in patients with disseminated melanoma.

### MATERIALS AND METHODS

*Patients.*—Sera were taken from patients with melanoma attending the Melanoma Unit, Sydney Hospital. H.I. and C.H. were patients with disseminated melanoma who had shown clinical improvement after surgery and chemo-immunotherapy and had no clinically detectable tumour. Patients J.S., C.Q., M.T., D.R., H.F. and B.N. had clinically evident disseminated melanoma. Patients B., R.G., N.D., R.L., D.T., M.C., A. and M. had primary melanoma which was subsequently removed surgically. Sera were from defibrinated blood samples and were stored at $-20°C$ in 2–3-ml-aliquots until use.

*Gel exclusion chromatography.*—Whole sera were separated into several fractions on the basis of molecular size using Sepharose CL-6B (Pharmacia) in a column $2{\cdot}6 \times 70$ cm (Type K26/70, Pharmacia). Two ml of whole serum was applied to the column and eluted with 0·9% saline containing 0·02% sodium azide by upward flow at a rate of 0·5 ml/min at 4°C. Fifty fractions of 6 ml each were collected.

U.v. absorbance of the fractions was measured at 280 nm on a Unicam SP1800 u.v. spectrophotometer, and the fractions were pooled into 4 groups according to molecular size, on the basis of chromatogram results. The large mol. wt fractions were concentrated back to 2 ml in an Amicon Diaflo cell, using an XM100-A membrane, and the small mol. wt fraction using a UM-2 membrane. They were then dialysed twice against 500 ml 0·9% NaCl. Immunoglobulins in each were identified by immunodiffusion on Tri-Partigen plates (Hoechst).

*Low-pH dissociation and ultrafiltration of whole sera.*—A modification of the method described by Sjögren *et al.* (1971) and de Schryver *et al.* (1976) was used in these studies. Serum (0·5 ml) was diluted 1:10 with Sorensen's glycine buffer, pH 2·8, and introduced into an Amicon Diaflo cell with an XM100-A membrane (retaining molecules $>$100,000 d). 45 ml buffer was added, dropwise, and allowed to stand at room temperature for 60 min. Filtration at 30 lb/in$^2$ was carried out to a volume of 1 ml and the pH of both solutions was adjusted to pH 7·2 with 0·9M $NaHCO_3$. The filtrate was then concentrated in a Diaflo cell on a UM-2 membrane (retaining molecules $>$1000 d) to 1–2 ml. Each fraction was then dialysed twice against 500 ml of 0·9% saline for 16–18 h, and the components identified by immunodiffusion on Tri-Partigen plates.

*$^{51}Cr$ release assays.*—The assay procedures used have been described in detail in previous reports (Hersey *et al.*, 1976*b*).

*Target cells (TCs).*—Melanoma cells from the continuous human line MM 200 were used for most of the studies. These, as well as the MM 127 and 170 lines used in the specificity studies, were kindly supplied by Dr J. Pope of the Queensland Institute of Medical Research. The antigens expressed on the surface of these cells have been described previously (Hersey *et al.*, 1976*b*). Chang cells were from the continuous human liver cell line (CSL, Melbourne). The ovarian carcinoma cells were established from ascitic fluid from metastatic ovarian carcinoma, and were kindly supplied by the Department of Surgery, University of Sydney. All TCs were grown in RPMI 1640 (Gibco) supplemented with 20% foetal bovine serum (FBS—Australian laboratory services, Batch 42) and were harvested by incubation with 0·25% trypsin for 15 min immediately before use.

*Effector cells.*—Mononuclear cells were obtained from defibrinated blood samples by centrifugation on Hypaque–Ficoll mixtures of sp. gr. 1·078 after methods described by Boyüm (1968). Unless otherwise stated, they were from healthy laboratory volunteers. The cells were resuspended in RPMI 1640 + 10% FBS at a concentration of $10^6$ ml for use in the assays.

*$^{51}Cr$ labelling.*—TCs were labelled with $^{51}Cr$ by incubation with 100 $\mu$Ci of $Na_2CrO_4$ (Amersham, Bucks, U.K.) for 2 h. The cells were washed twice in 25 ml of Eagles minimal essential medium (MEM) (CSL, Melbourne) and resuspended in RPMI + 10% FBS at a concentration of $10^4$/ml for use in the assays.

*LDA assays.*—Fractions obtained by the chromatography procedures described, or by low-pH dissociation and ultrafiltration, were assayed for LDA by addition of 100 $\mu$l of the fraction at the dilutions indicated to cultures containing $5 \times 10^3$ in 500 $\mu$l of $^{51}$Cr-labelled TCs (usually MM 200). This was followed by the addition of $5 \times 10^5$ effector cells in 500 $\mu$l. The cultures were in duplicate (12 $\times$ 75) mm round-bottomed plastic tubes (Sterilin, Filtrona, Melbourne). The tubes were capped and incubated overnight for 16 h at 37°C in 7% $CO_2$ in air. Assays were terminated by centrifugation for 7 min at 400 *g*, and 500 $\mu$l of the supernatant removed for estimation of $^{51}$Cr release. Samples were counted in an automatic Wallac 1280 gamma counter.

Percent $^{51}$Cr release was estimated by the formula

$$\frac{a \times 2{\cdot}2}{a + b} \times 100$$

where a = $^{51}$Cr counts in the supernatant-background count, and b = counts in tube with remaining supernatant and cells-background counts. The LDA titre of sera or fractions of sera was taken as the last dilution giving 5% $^{51}$Cr release above the baseline release of target cells and effector cells in the absence of added sera or sera fractions. This level of $^{51}$Cr release was about 2 standard errors from the points concerned, and was taken as the minimum definite evidence of LDA activity in the test.

*Blocking of LDA assays.*—Sera were tested for blocking of LDA activity by addition to cultures containing IgG known to induce LDA reactivity against melanoma cells. 100 $\mu$l of the serum to be tested for blocking activity was added to the TCs, followed by 100 $\mu$l of the sensitizing IgG antibody and $5 \times 10^5$ effector cells in 500 $\mu$l. Incubation was carried out overnight and percent $^{51}$Cr release estimated as above, except that the multiplication factor for "a" was 2·4. The percentage inhibition or blocking of the antibody-dependent killing was estimated by the formula

$$\frac{A - B}{A} \times 100$$

where A = % $^{51}$Cr release induced by IgG LDA above baseline of TC + effectors alone, B = % $^{51}$Cr release induced by IgG LDA above baseline of TC + effectors with added test blocking material. (Where the added test fractions had LDA activity, the baseline was taken as for A above).

In experiments to test for affinity of blocking activity to target cells, or inhibitory activity to effector cells, 100 $\mu$l of the test material was added to either target cell or effector cell for 1 h at room temperature, and the tubes were then washed twice in 5 ml of MEM. The tubes were drained and 100 $\mu$l of sensitizing IgG melanoma antibody added, together with the effector cells in 1 ml of medium. In these experiments control cultures not exposed to the test material were subjected to a similar washing procedure in parallel with the test cultures.

*Statistical analysis.*—Student's *t* test was used to compare the percent $^{51}$Cr release of cultures with and without LDA activity, and to compare LDA activity in the presence or absence of added blocking factors. Findings were considered significant when $P < 0{\cdot}05$.

### RESULTS

### *LDA activity against melanoma cells of whole sera and IgG fractions*

Fig. 1 illustrates a representative assay of LDA activity of whole serum and the IgG fraction of serum from patient G.Q.

Fig. 1.—LDA activity of unfractionated serum and the 7s fraction of serum from a melanoma patient against MM 200 melanoma target cells and control non-melanoma "Chang" cells. LDA activity to melanoma cells is shown in the 7s fraction but not the whole serum. TC = spontaneous $^{51}$Cr release from target cell alone; TC + Eff = $^{51}$Cr release from target cell due to natural cytotoxicity of the effector cells; (s.e. mean < 2%).

with disseminated melanoma, against melanoma cells from the MM 200 line and the control Chang cell line. No LDA activity was seen in the unfractionated serum against the melanoma cell but the IgG fraction had a titre of $1/10^2$ to the melanoma cells but not the control Chang cells. In Table I similar studies are shown in 8 patients with disseminated melanoma and 8 with primary melanoma. Seven of the 8 patients with disseminated melanoma had LDA activity in the IgG fraction but not in their unfractionated sera. Four patients with primary melanoma had activity in the IgG fraction but not in their unfractionated sera. The other 4 primary melanoma patients had no detectable LDA activity in either the IgG fraction or the unfractionated sera. No sera from 7 control normal subjects or 6 non-melanoma subjects had LDA activity in the IgG fraction against the MM 200 cell line. Negative data from sera of 2 patients with carcinoma of the breast are not shown in the Table. (A.E. had activity in both her whole serum and the IgG fraction. Previous studies (Hersey *et al.*, 1976*b*) have suggested this activity is against foetal antigens on the melanoma cells. None of the sera or fractions of sera from the melanoma patients tested had detectable activity against the control Chang cell. Serum A.E. reacted with Chang cells to a titre of $\frac{1}{10}$. Serum and serum fractions from melanoma patients H.I., G.Q. and M.C. did not have LDA activity against the ovarian carcinoma cells. None of the sera or serum fractions in these studies had any effect on $^{51}Cr$ release when cultured alone with the target cells in the absence of effector cells.

### *LDA reactivity of melanoma sera after low-pH dissociation and ultrafiltration*

Figure 2 illustrates the LDA activity of serum from Melanoma Patient G.Q. and serum fractions obtained from the whole serum after lowering the pH to 2·8 and

FIG. 2.—LDA activity of unfractionated serum from melanoma patient together with that of the retentate (A) and filtrate (B) obtained after acidification to pH 2·8 and ultrafiltration through membranes retaining molecules greater than 100,000 d. Activity is seen in retentate but not serum, filtrate or combination of filtrate and retentate. TC and TC + Eff are as described in legend of Fig. 1. (s.e. mean < 2%).

TABLE I.—*Comparison of LDA Activity to Melanoma Cells of Unfractionated Sera and the IgG-containing Fraction of Sera*

| Non-melanoma subjects | Whole serum | IgG fraction | Subjects with disseminated melanoma | Whole serum | IgG fraction | Subjects with primary melanoma | Whole serum | IgG fraction |
|---|---|---|---|---|---|---|---|---|
| E.S. | 0 | 0 | C.H. | 0 | $10^2$ | B. | 0 | 0 |
| H.B. | 0 | 0 | H.I. | 0 | $10^2$ | N.D. | 0 | 10 |
| B.M. | 0 | 0 | G.Q. | 0 | $10^2$ | B.G. | 0 | 0 |
| J.Z. | 0 | 0 | H.F. | 0 | 0 | R.L. | 0 | 10 |
| A.E. | $10^2$ | $10^2$ | M.T. | 0 | 10 | D.T. | 0 | 10 |
| S.C. | 0 | 0 | J.S. | 0 | 10 | M.C. | 0 | $10^2$ |
| P.M. | 0 | 0 | G.M. | 0 | 10 | A. | 0 | 0 |
| L.P.* | 0 | 0 | D.R. | 0 | 10 | M. | 0 | 0 |
| P.P.* | 0 | 0 | | | | | | |
| J.G.* | 0 | 0 | | | | | | |
| J.W.* | 0 | 0 | | | | | | |

* Carcinoma other than melanoma (stomach, basal-cell carcinoma, lung and oesophagus, respectively).

Values indicated are reciprocal dilutions of sera or IgG fractions of sera, giving $^{51}Cr$ release 5% above baseline from target cell and effector cells alone.

Footnote: Spontaneous $^{51}Cr$ release from TCs ranged from 22 to 38%. $^{51}Cr$ release due to cytotoxicity of effector cells ranged from 5 to 15% above spontaneous release (see Fig. 1).

ultrafiltration through an Amicon filter retaining molecules greater than 100,000d. Activity was seen in the retentate but not in the whole serum or filtrate. Recombination of filtrate and retentate resulted in blocking of the activity of the retentate. The retentate did not have LDA activity against the control Chang cell.

The results in Table II indicate similar studies on sera from 4 patients with disseminated melanoma, 2 normal subjects and 4 non-melanoma carcinoma subjects. All of the melanoma sera had LDA activity in the retained fraction after dissociation and ultrafiltration, but sera from the other subjects did not. Also illustrated in the Table are control studies showing that no LDA activity was seen when the ultrafiltration was carried out after restoration of the pH to 7·4, or when saline instead of the glycine buffer at pH 2·8 was added to the serum. Only slight activity was seen when ultrafiltration of the acidified serum was carried out through membranes retaining molecules greater than 10,000d, indicating that the blocking factors were between 10,000 and 100,000d.

TABLE II.—*LDA Reactivity of Melanoma Sera, Before and After Low-pH Dissociation and Ultrafiltration*

| | Titre of LDA reactivity | | | |
|---|---|---|---|---|
| Subject | Whole serum | Retained fraction | Filtrate | Recombined fractions |
| G.M. | 0 | $10^2$ | 0 | 10(50) |
| D.R. | 0 | 10 | 0 | 10(50) |
| H.I. | 0 | $10^2$ | 0 | 10(29) |
| M.T. | 0 | 10 | 0 | 0(75) |
| M.T. (1) | 0 | 0 | 0 | 0 |
| M.T. (2) | 0 | 0 | 0 | 0 |
| M.T. (3) | 0 | 0 | 0 | 0 |
| E.A.* | 0 | 0 | 0 | 0 |
| P.M.* | 0 | 0 | 0 | 0 |
| (a) G. | 0 | 0 | 0 | 0 |
| (b) Pe | 0 | 0 | 0 | 0 |
| (c) W | 0 | 0 | 0 | 0 |
| (d) Pi | 0 | 0 | 0 | 0 |

Figures in brackets represent % inhibition of $^{51}Cr$ release above the baseline of TC and effector cells alone, on recombination of the two fractions.

* Normal subjects

(a) Carcinoma of lung, (b) stomach, (c) oesophagus, and (d) skin

(1) pH restored to 7·4 before ultrafiltration, (2) Ultrafiltration through PM-10 instead of XM-100 membranes, (3) Ultrafiltration after addition of saline instead of glycine buffer at pH 2·8

See also footnote to Table 1.

*Titration of blocking activity and affinity of blocking activity for target cells or effector cells*

Experiments were conducted to determine the titre of blocking activity and whether the blocking activity was directed to the target cell or the effector cells. One such experiment is illustrated in Fig. 3, in which serum from Subject G.Q. was added to MM 200 TCs sensitized with a constant amount of the IgG fraction from G.Q. Significant blocking activity was still present at a titration of $1/10^4$ of the serum when present throughout the culture period. When the blocking serum was added to the target cells and washed, marked inhibition of the LDA activity of added IgG was still shown. Blocking was less marked however when the serum was added to the effector cells and washing carried out.

Similar results to those illustrated in Fig. 3 are shown in Table III. In all experiments, the blocking activity of the sera showed affinity for the target cell

FIG. 3.—(A) Titration of blocking activity of melanoma serum against melanoma IgG LDA activity. (B, C) Blocking activity of serum produced by preincubation with TC, (B) effector cell (C) following by washing. The sensitizing IgG LDA, and effector (B) or target cells (C) were then added. (Target cells from MM 200 melanoma cell line.) B.S. = blocking serum. TC and TC + Eff are as described in legend to Fig. 1. IgG refers to LDA activity of IgG fraction from melanoma subject G.Q. In (B) and (C) blocking serum was added at a dilution of $\frac{1}{10}$ (s.e. mean < 2%).

and little or no blocking was seen when preincubated with the effector cells.

*Specificity of the blocking activity of melanoma*

To determine the broad specificity of the blocking activity to melanoma, sera from patients with disseminated melanoma were added to cultures of Chang cells sensitized with rabbit anti-Chang serum (Hersey, Edwards and Edwards, 1976). As shown in Table IV, no blocking of the killing of sensitized Chang cells by leucocytes was seen. This applied even when the IgG fraction from the melanoma patient was present as well.

Initial studies to determine the specificity of the blocking activity between different melanoma patients are also shown in Table IV. Blocking serum from B.N. did not block the IgG LDA from J.S. when the TC was MM 170, but did when the TC was MM 127. M.T. serum showed partial blocking of B.N. IgG LDA.

### DISCUSSION

From these initial studies it appears that masking of LDA activity is a common finding in the sera of patients with advanced melanoma, and to a lesser extent in patients with primary melanoma. This has been demonstrated by several methods, including separation of the IgG fraction from sera by gel chromatography, and acidification and ultrafiltration of melanoma sera to dissociate and separate immune complexes, similar studies to those previously described by Sjögren *et al.* (1971, 1972). In view of the suggested biological importance of LDA in tumour rejection (Lamon *et al.*, 1973; Hersey, 1973; O'Toole *et al.*, 1974) the presence of these blocking factors against LDA may be an important factor in allowing escape of the tumour cells from this immune cytotoxic mechanism.

The specificity of the LDA activity detected in these sera has not yet been extensively studied. However, none of the melanoma sera or sera fractions reacted with the control Chang liver cell, and 3 of the strongly positive 7s fractions from melanoma sera did not react with ovarian carcinoma cells in culture. Perhaps more importantly, we have not shown LDA activity in the IgG-containing fractions of sera from 7 normal subjects and 6 patients with various malignancies other than melanoma. This suggests that the LDA activity revealed in the 7s fractions of melanoma sera is related to melanoma. From our previous studies on the specificity of LDA found in melanoma sera, some reactivity against non-melanoma cells may be expected on the basis of cross-reacting foetal antigens (Hersey *et al.*, 1976*b*).

Several other questions arise from these studies. One concerns the nature of the blocking activity detected in these assays.

TABLE III.—*Affinity of Blocking Activity for Target or Effector Cells*

| Subject | IgG LDA | IgG LDA in presence of whole serum | Effect on IgG LDA activity of pre-incubation of TC | Effect on IgG LDA activity of pre-incubation of effector cells |
|---|---|---|---|---|
| G.Q. | 13 | 1* (92) | 2* (83) | 8 (38) |
| C.H. | 10 | 0* (100) | 5* (50) | 10 (–) |
| M.T. | 18 | 4* (78) | 3* (83) | 16 (11) |

Values indicated are % $^{51}$Cr release induced by IgG LDA above baseline due to cytotoxicity of AD effector cells in absence or presence of serum blocking factors. (Maximum s.e. mean of the points was 2%.)

* $P < 0{\cdot}05$ for difference between IgG LDA activity in absence and presence of blocking serum.

Figures in brackets are the percentage blocking activity of the IgG LDA produced by the whole serum in terms of inhibition of percent $^{51}$Cr release above the baseline.

See also footnote to Table I.

TABLE IV.—*Specificity of Serum Blocking Activity against Melanoma LDA*

| Blocking serum | TC | Sensitizing IgG LDA | % $^{51}Cr$ release above baseline induced by LDA: No blocking serum | Blocking serum† |
|---|---|---|---|---|
| M.T. | MM 200 | M.T. | 12 | 5* |
| C.H. | MM 200 | C.H. | 12 | 4* |
| J.S. | MM 200 | J.S. | 6 | 1* |
| H.I. | MM 200 | H.I. | 25 | 1* |
| P.M.** | MM 200 | E.H. | 15 | 14 |
| L.S.** | MM 200 | E.H. | 15 | 14 |
| M.T. | Chang | Rabbit anti-Chang | 11 | 11 |
| C.H. | Chang | Rabbit anti-Chang | 8 | 10 |
| C.H. | Chang | Rabbit anti-Chang + C.H. | 8 | 8 |
| J.S. | Chang | Rabbit anti-Chang | 8 | 10 |
| J.S. | Chang | Rabbit anti-Chang + J.S. | 8 | 9 |
| J.S. | MM 170 | J.S. | 13 | 5* |
| B.N. | MM 170 | J.S. | 13 | 13 |
| M.T. | MM 127 | B.N. | 8 | 4 |
| B.N. | MM 127 | J.S. | 10 | 4* |

* $P < 0 \cdot 05$.
** Normal subjects.
† 1/10 final dilution.
(Maximum s.e. mean of the values was 2% $^{51}Cr$ release.) See also footnote to Table I.

In common with the findings of Sjögren *et al.* (1971, 1972) in studies on blocking of direct CMC to tumour cells, we have found that the blocking activity against melanoma LDA can be dissociated into large- and small-mol.-wt components and that the small-mol.-wt component was between 10,000 and 100,000 daltons. This evidence suggested that immune complexes were involved and that the small-mol.-wt component may be tumour antigen. The size of the antigen in these studies would be consistent with that described for melanoma antigens by Currie (1973).

The second question relating to these studies is the specificity of the blocking activity. The results above indicate that the blocking activity in the sera tested was directed to the target cell and not the effector cells. This was further shown by the studies in which no blocking was seen when the sera were added to LDA assays of Chang cells sensitized with rabbit anti-Chang serum. This latter test system was shown previously to be inhibited non-specifically by immune complexes interacting with the effector cells (MacLennan, 1972). It was found in these studies that complexes in slight antigen excess showed maximum inhibition, and that complexes in antibody excess had little or no inhibitory activity. Our results therefore suggest that if immune complexes are responsible for the blocking activity they are probably in antibody excess. This would explain the absence of non-specific blocking of LDA activity and the specificity of the blocking activity for the target cells. If this is so, it can be expected that the specificity of blocking will be similar to that described in our previous studies (Hersey *et al.*, 1976*b*) in which limited cross-reactivity of antisera between different individuals with melanoma was found.

The findings above appear to provide an analytical assay system which we anticipate may be useful in further characterizing blocking factors. Preliminary results of work in progress also suggest that the measurement of blocking activity against melanoma LDA may be of value in monitoring patients with melanoma.

This work was supported by the University of Sydney Cancer Research Fund, the N.S.W. State Cancer Council and the Bill White Melanoma Fund. We wish to thank Mrs A. Edwards and Mrs E. Adams for assistance with these studies.

### REFERENCES

BALDWIN, R. W., EMBLETON, M. J. & PRICE, M. R. (1973) Inhibition of lymphocyte Cytotoxicity for Human Colon Carcinoma by Treatment with Solubilized Tumour Membrane Fractions. *Int. J. Cancer*, **12**, 84.

BALDWIN, R. W., PRICE, M. R. & ROBINS, R. A. (1972) Blocking of Lymphocyte-mediated Cytotoxicity for Rat Hepatoma Cells by Tumour-specific Antigen–Antibody Complexes. *Nature, New Biol.*, **238**, 185.

BALDWIN, R. W., PRICE, M. R. & ROBINS, R. A. (1973) Significance of Serum Factors Modifying Cellular Immune Responses to Growing Tumours. *Br. J. Cancer*, **28**, Suppl. 1, 37.

BANSAL, S. C. & SJÖGREN, H. O. (1971) "Unblocking" Serum Activity *in vitro* in the Polyoma System may Correlate with Antitumour Effects of Antiserum *in vivo*. *Nature, New Biol.*, **233**, 76.

BANSAL, S. C. & SJÖGREN, H. O. (1973) Regression of Polyoma Tumour Metastasis by Combined Unblocking and BCG Treatment—Correlation with Induced Alterations in Tumour Immunity Status. *Int. J. Cancer*, **12**, 179.

BÖYUM, A. (1968) Isolation of Mononuclear Cells and Granulocytes from Human Blood. *Scand. J. clin. Lab. Invest.*, **21**, 77.

CURRIE, G. (1973) The Role of Circulating Antigen as an Inhibitor of Tumour Immunity in Man. *Br. J. Cancer*, **28**, Suppl. 1, 153.

CURRIE, G. A. & BASHAM, C. (1972) Serum mediated Inhibition of the Immunological Reactions of the Patient to his Tumour. A Possible Role for Circulating Antigen. *Br. J. Cancer*, **26**, 427.

DE SCHRYVER, A., ROSEN, A., GUNVEN, P. & KLEIN, G. (1976) Comparison between Two Antibody Populations in the EBV System: Anti-MA *versus* Neutralizing Antibody Activity. *Int. J. Cancer*, **17**, 8.

HELLSTRÖM, I. & HELLSTRÖM, K. E. (1969) Studies on Cellular Immunity and its Serum mediated Inhibition in Moloney Virus induced Mouse Sarcoma. *Int. J. Cancer*, **4**, 587.

HELLSTRÖM, I. & HELLSTRÖM, K. E. (1973) Some Recent Studies on Cellular Immunity to Human Melanoma. *Fed. Proc.*, **32**, 156.

HELLSTRÖM, I., SJÖGREN, H. O., WARNER, G. & HELLSTRÖM, K. E. (1971) Blocking of Cell-mediated Tumor Immunity by Sera from Patients with Growing Neoplasms. *Int. J. Cancer*, **7**, 226.

HELLSTRÖM, I., WARNER, G. A., HELLSTRÖM, K. E. & SJÖGREN, H. O. (1973) Sequential Studies on Cell mediated Tumor Immunity and Blocking Serum Activity in Ten Patients with Malignant Melanoma. *Int. J. Cancer*, **11**, 280.

HERSEY, P. (1973) A New Look at Antiserum Therapy of Leukaemia. *Nature, Lond.*, **244**, 22.

HERSEY, P., EDWARDS, A. E. & EDWARDS, J. (1976) Characterization of Effector Cells in Human Blood. *Clin. exp. Immunol.*, **23**, 104.

HERSEY, P., HONEYMAN, M., EDWARDS, A., ADAMS, E. & McCARTHY, W. H. (1976*b*) Antigens on Melanoma Cells Detected by Leukocyte dependent Antibody Assays of Human Melanoma Antisera. *Int. J. Cancer*, **18**, 564.

HERSEY, P., ISBISTER, J., EDWARDS, A., MURRAY, E., ADAMS, E. & MILTON, G. W. (1976*a*) Antibody dependent Cell mediated Killing of Melanoma Cells Induced by Plasmapheresis. *Lancet*, i, 825.

JOSE, D. G. & SESHADRI, R. (1974) Circulating Immune Complexes in Human Neuroblastoma: Direct Assay and Role in Blocking Specific Cellular Immunity. *Int. J. Cancer*, **13**, 824.

LAMON, E. W., WIGZELL, H., KLEIN, E., ANDERSSON, B. & SKURZAK, H. M. (1973) The Lymphocyte Response to Primary Moloney Sarcoma Virus Tumours in BALB/c Mice. Definition of the Active Subpopulations at Different Times after Infection. *J. exp. Med.*, **137**, 1472.

MACLENNAN I. C. M. (1972) Competition for Receptors for Immunoglobulin on Cytotoxic Lymphocytes. *Clin. exp. Immunol.*, **10**, 275.

O'TOOLE, C., STEJSKAL, V., PERLMANN, P. & KARLSSON, M. (1974) Lymphoid Cells Mediating Tumor-specific Cytotoxicity to Carcinoma of the Urinary Bladder. *J. exp. Med.*, **139**, 457.

SJÖGREN, H. O., HELLSTRÖM, I., BANSAL, S. C. & HELLSTRÖM, K. E. (1971) Suggestive Evidence that the "Blocking Antibodies" of Tumour-bearing Individuals may be Antigen–Antibody Complexes. *Proc. natn. Acad. Sci., U.S.A.*, **68**, 1372.

SJÖGREN, H. O., HELLSTRÖM, I., BANSAL, S. C., WARNER, G. A. & HELLSTRÖM, K. E. (1972) Elution of Blocking Factors from Human Tumours Capable of Abrogating Tumour Cell Destruction by Specifically Immune Lymphocytes. *Int. J. Cancer*, **9**, 274.

THOMPSON, D. M. P., ECCLES, S. & ALEXANDER, P. (1973) Antibodies and Soluble Tumour-specific Antigens in Blood and Lymph of Rats with Chemically Induced Sarcomata. *Br. J. Cancer*, **28**, 6.